# Generalized pustular psoriasis (von Zumbusch)^☆^

**DOI:** 10.1016/j.abd.2021.05.011

**Published:** 2021-11-24

**Authors:** Ricardo Romiti, André Luís da Silva Hirayama, Marcelo Arnone, Renata Ferreira Magalhães

**Affiliations:** a Department of Dermatology, Hospital das Clínicas da Faculdade de Medicina da Universidade de São Paulo, São Paulo, SP, Brazil; b Discipline of Dermatology, Faculdade de Ciências Médicas, Unicamp, Campinas, SP, Brazil

**Keywords:** Acrodermatitis, Psoriasis, Psoriasis/epidemiology, Psoriasis/genetics, Psoriasis/physiopathology, Psoriasis/therapy

## Abstract

Generalized pustular psoriasis (von Zumbusch) is a rare and acute eruption characterized by multiple sterile pustules over an erythematous and edematous background, eventually associated with psoriasis vulgaris. Classically, it manifests as a potentially severe systemic picture and demands prompt diagnosis and intervention. The duration of each flare-up and intervals between the pustular episodes is extremely variable. Recently, genetic abnormalities have been identified mainly in the familial and early variants of this disease. The therapeutic arsenal is limited; however, new drugs being evaluated aim to control both pustular flare-ups and disease recurrences.

## Introduction

Inflammatory conditions that progress with sterile pustules on an erythematous background, with variable topography and extension, and that reflect neutrophil infiltration in the epidermis are characteristic of the pustular form of psoriasis. This variant may arise from or coexist with chronic plaque psoriasis lesions or arise and recur in the absence of any other form of psoriasis. It might present as a severe and life threatening disorder.^1^

There are four subtypes of pustular psoriasis: 1) generalized pustular psoriasis (GPP), von Zumbusch, 2) annular or circinate pustular psoriasis, 3) exanthematic pustular psoriasis, and 4) localized pustular psoriasis, including the palmoplantar pustular psoriasis (PPPP) and acrodermatitis continua of Hallopeau (ACH) variants. Mixed variants have also been described.^1^

GPP is an acute and severe multisystem variant of psoriasis and manifests as erythematous and edematous plaques on which multiple pustules appear, associated or not with the plaque form. In this review, the main aspects of GPP will be addressed in relation to the epidemiology, genetics, pathophysiology, clinical picture, therapy, and prognosis of this rare entity.

## Epidemiology

Since the original report by Leo Ritter von Zumbusch (Vienna, 1874–1940) in 1909,^2^ describing the occurrence of GPP in siblings as well as disease evolution, the classification of GPP as the most severe variant of plaque psoriasis or as a distinct clinical entity has been discussed.^1^

The estimated prevalence of GPP is higher in the Asian ethnicity (7.46 per million in Japan)^3^ than in Caucasian populations (1.76 per million in France).^4^ It is more prevalent in the female sex and can appear in any age group, being more frequent in adults between the fourth and fifth decades of life.^3^, ^4^, ^5^ Registry data with 15,794 individuals with psoriasis in the Asian population in Malaysia estimated the prevalence of GPP as around 1%,^6^ while an epidemiological study in Japan reported an occurrence of 1.3% of a total of 11,631 cases of psoriasis.^7^ The association with plaque psoriasis has been reported as ranging from 25%–30% to 65% of cases.^8^, ^9^ In the pediatric population, it seems to occur more frequently in the male sex (3:2), ranging from 0.6 to 7% of psoriasis cases.^5^, ^10^

Currently, GPP is essentially considered to be a distinct entity from plaque psoriasis, with a distinct genetic and pathogenic profile.^5^, ^11^ Hussain et al. found that in patients with a mutation in the interleukin-36 receptor antagonist (IL-36RN) gene, the onset of GPP tends to occur earlier, associated with a higher risk of systemic inflammation and a lower association with plaque psoriasis.^12^

## Genetics

The genetic basis of plaque psoriasis is evident in the higher prevalence of the disease among first-degree relatives, two to three-fold higher than in the general population. Many genomic scanning studies replicated in different populations have demonstrated genetic polymorphisms associated with plaque psoriasis.^13^ There are more than 50 chromosomal regions or loci of susceptibility to psoriasis, demonstrating the multigene and epigenetic interaction characteristic of the disease.^14^ The genes whose polymorphisms confer susceptibility to plaque psoriasis are related to antigen presentation (ERAP1 and MHC), interferon signaling pathway (IL-28RA and IFIH1), NFkB pathway (ZNF313, REL, TNIP1, TNFAIP3, NFkB1A),^15^ of IL-17 (TRAF3IP2), of IL-23 (TYK2, IL-23R, IL-23A, IL-12B) and to the epidermal barrier (LCE3D).^16^

Responsible for more than 50% of the inheritability of the disease, the genes of the main histocompatibility complex (MHC) that encode membrane human leukocyte antigens (HLA) are located in a genomic segment at position 6p.21, with approximately 15 other genes. The susceptibility allele most closely related to psoriasis is HLA-Cw*0602. Class I Histocompatibility Complex (HLA) molecules recognize non-self-antigens and present them to CD8+ T lymphocytes to initiate an immune response and favor signaling between different cell types.^16^

On the other hand, genetic studies of the various forms of pustular psoriasis reveal differences when compared with plaque psoriasis. HLA polymorphisms and epidermal barrier genes do not confer susceptibility to this form of the disease. However, mutations in genes related to innate immunity, such as IL-36RN, AP1S3, and CARD14, are important in some patients.^17^

The analysis of 5,249 patients with different forms of pustular psoriasis showed that IL36RN mutations are the most frequently observed genetic abnormality in pustular psoriasis.^18^ The genes encoding the IL-36 family are located on chromosome 2q13.^18^

Autosomal recessive loss-of-function mutations in the IL-36RN gene are found in approximately 25% of GPP cases. This gene encodes the IL-36 receptor antagonist (IL-36Ra), which modulates the activity of IL-1 family cytokines (IL-36α, -β and -γ). Genotype-phenotype analyses indicate that mutated alleles are less common in individuals with PPPP than GPP and ACH, and when there is a single mutation in the IL-36RN gene, the disease tends to manifest later in life. Variable penetrance of the alleles and genetic modifiers occurs, and environmental factors also influence their expression.^19^ Homozygous mutations in the IL-36RN gene are associated with earlier age of onset in all variants of pustular psoriasis, more severe disease evolution, and different therapeutic responses when compared to plaque psoriasis.^18^

The AP1S3 gene (adaptor-related protein complex 1, Sigma-3 subunit) is located at chromosomal position 2q36.1. Adapter protein (AP) complexes are cytosolic heterotetramers that promote the production and mobilization of small transport vesicles. AP-1 is dedicated to the transportation between the trans-Golgi network and the endosomes. The Sigma-3 subunit is the main stabilizer of AP-1 and is encoded by the AP1S3 gene. AP-1 is responsible for the formation of autophagosomes, intracellular structures for protein, and damaged organelle degradation. Defective autophagy leads to accumulation of p62 (an adapter protein in NF-kB activation) and upregulation of inflammation by IL-36.^19^ Mutations in the AP1S3 gene were found in 7 to 12% of European patients. In the study by Twelves et al., mutations in AP1S3 were found with comparable frequency across disease types. The known mutations are p.Phe4Cys and p.Arg33Trp.^18^

The CARD14 gene (caspase recruitment domain-containing protein 14, also called CARD-containing MAGUK protein 2 or Carma 2) is located at chromosomal position 17q25.3. It is highly expressed in keratinocytes and encodes a protein that, after oligomerization, mediates the activation of NF-κB signaling by TRAF-2, being responsible for the ordering of this activation and of the MAPK signaling pathways through MALT and BCL10 recruitment. It leads to the stimulation of pro-inflammatory genes IL-36, IL-8, CCl20, and antimicrobial peptides.^20^, ^21^ A gain-of-function substitution in CARD14 has been associated with GPP. They have also been described in plaque psoriasis and palmoplantar pustulosis, indicating shared mechanisms. CARD14 expressed in keratinocytes associates with the ACT1-TRAF6 signaling complex and mediated the activation of the IL-17A-induced NF-κB and MAPK signaling pathway, which leads to the expression of pro-inflammatory factors. Thus, it is also a key mediator of IL-17A signaling.^18^

In the study by Twelves et al., CARD14 mutations were observed in only eight patients. Additionally, the only disease allele associated with pustular (p.Asp176His) or plaque psoriasis (p.Gly117Ser) has been found in patients with GPP of Chinese descent and has not yet been detected in European patients.^18^

This study also aimed to evaluate the clinical and genetic characteristics of pustular psoriasis in a cohort of unrelated patients (251 with GPP, 560 with PPPP, 28 with APH, and 24 with multiple diagnoses). The mutated IL-36RN alleles were present in a variety of ethnic groups, with the highest prevalence being observed among patients of European (34.7%) and Asian (28.8%) descent, and it was highest in GPP and APH (23.7 % and 18.2%, respectively) when compared to PPPP (5.2%). An analysis of the recurrent variant p.Ser113Leu showed that its frequency in British patients was almost 10-fold higher than that seen in a control population.^18^ This variant has also been reported in two cases of Brazilian patients.^22^ AP1S3 alleles had a similar and low frequency in all disease subtypes. Risk alleles at two distinct loci (IL-36RN and AP1S3; IL-36RN and CARD14) have been reported in several cases, showing that genetic heterogeneity is complex and pleiotropysm and digenic inheritance can influence disease expression.^18^

## Immunogenetics (Table 1)

The GPP transcriptome is highly associated with genes of the innate immune system, although it shares characteristics of plaque psoriasis.^18^, ^23^ The expression levels of IL-1β, IL-1RN, IL-36α, IL-36β, IL-36γ and IL-36Ra increase in GPP and plaque psoriasis when compared to normal skin, being more intense in the pustular form.^18^, ^23^ Increased signaling for IL-1 and IL-36 in GPP is related to a significant increase in the expression of chemotactic substances to neutrophils (CXCL1, CXCL2 and CXCL8).^23^

**Table 1:** Explanatory glossary of terms involved in the immunogenetics of pustular psoriasis.

Terms	Definition
NFkB	It comprises a sequence of 11 base pairs that interacts with the light chain of the immunoglobulin produced by B lymphocytes, a protein complex that performs functions as a transcription factor, i.e., activated in the cell membrane, it goes to the nucleus to order the transcription of specific genes. The Kappa B Nuclear Factor (NFκB) is a family of transcription factors vital to the coordination of inflammatory responses of the innate and adaptive immune systems, cell differentiation, proliferation and survival in almost all multicellular organisms.^15^, ^20^
BCL10	The BCL10 gene (B-cell CLL/lymphoma 10) encodes a protein complex member (CBM), which also contains adapters from the CARD family, for the recruiting of caspases such as CARD9 and MALT1. The CBM complex is involved in NFkB activation after stimulation of several receptors on lymphoid, myeloid and epithelial cells. BCL10 forms heterotrimers with different CARD proteins in different cell types.^20^
MAPK	The MAPK (mitogen-activated protein kinase) gene encodes the enzyme participating in intracellular signaling pathways.^20^
MALT1	The MALT1 (mucosa-associated lymphoid tissue lymphoma translocation gene 1) gene or paracaspase encodes a caspase-like cysteine protease essential for the activation of kappa-B nuclear factor downstream of cell surface receptors.^20^
CXCL	Chemokines comprise a group of small molecules (approximately 8 to 14 kD) that regulate several types of leukocytes through interactions with a subset of 7-transmembrane G protein-coupled receptors. They are divided into two main subfamilies, CXC and CC, based on the arrangement of the first two of the four conserved cysteine residues; the two cysteines are separated by a single amino acid in the CXC chemokines and are adjacent in the CC chemokines. CXC chemokines are subdivided into ELR and non-ELR types, based on the presence or absence of an adjacent glu-leu-arg sequence and N-terminal. ELR types are chemotactic for neutrophils, while non-ELR types are chemotactic for lymphocytes.^20^ For example, IL-8 is known as chemokine (*C-X-C* motif*)* ligand 8, CXCL8.
Inflammasome	The innate immune system has pattern recognition receptors (PRRs) for specific molecular sequences present in microorganisms and conserved during evolution, called pathogen-associated molecular patterns (PAMPs). Toll-like receptors (TLRs) and nucleotide-binding receptors containing repeated sequence domain of leucine amino acid residues (NLRs) are considered to be the main PAMP recognition receptors. TLRs also recognize endogenous molecules called damage-associated molecular patterns (DAMPs), which are released during stress and cell damage. The activation of these receptors increases the expression of genes and factors that control the production of type I interferon and pro-inflammatory cytokines. While TLRs are expressed in the cell membrane, NLRs are cytoplasmic sensors that oligomerize to form an activating protein complex, which is the inflammasome.^46^

**Figure 1:**
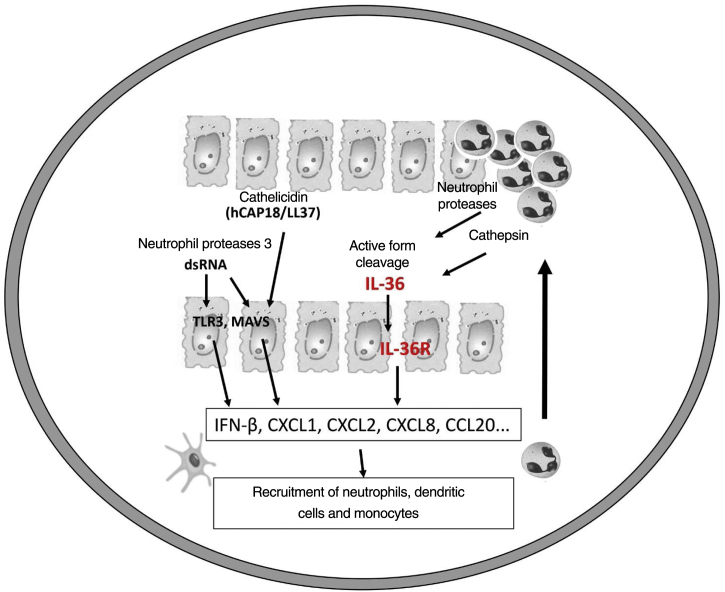
Pathogenesis of pustular psoriasis and activation of the IL-36 pathway.

It is believed that an initial trigger is the activation of DC plasmacytoid, stimulated by complexes of host DNA debris and LL-37, which are produced by keratinocytes after injury. Human cathelicidins (hCAP18/ LL-37) are antimicrobial peptides, and active LL-37 is released from the proform hCAP18 through proteolysis exerted by proteinase interacting with toll-like receptors for the activation of dendritic cells (Fig. 1).^23^

Epithelial cells, including keratinocytes, are sources of cytokines of the IL-1 family (IL-1F), consisting of 11 members with its most common nomenclature (and alternative names in parentheses): IL-1α, IL-1β, receptor antagonist of IL-1 (IL-1RN), IL-18, IL-33, IL-36α (IL-IF6), IL-36β (IL-1F8), IL-36γ (IL-1F9), IL-36Ra (IL-1F5), IL-37 (IL-1F7) and IL-38 (IL-1F10).^24^

**Figure 2:**
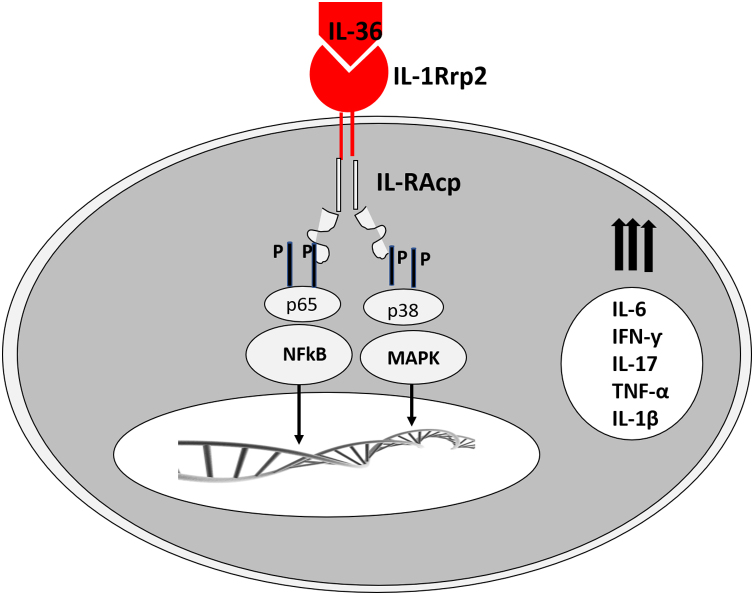
IL-36 action on its receptor and activation of intracellular pathways for the activation of transcription of pro-inflammatory genes.

The corresponding receptors are named as follows: for IL-36α and β, IL-1Rrp2 and IL-1RAcp receptors, expressed in monocytes, T and B lymphocytes; for IL-36γ, the receptors are the same but expressed in keratinocytes and epithelial cells; for IL-36Ra, the receptors are IL-1Rrp2 and SIGIRR, expressed by keratinocytes, monocytes, and dendritic cells.^18^ Members of IL-36 use the same IL-36R receptor, with the first three showing similar levels of agonist activity after binding, but the binding to IL-36Ra does not initiate a signaling response; therefore, it is considered antagonistic (Fig. 2).^23^, ^24^

The expression of IL-36γ was located in the granular layer of the epidermis, especially in peripustular keratinocytes. The IL-36α expression has also been strongly detected in the superficial layers of the epidermis in GPP lesions and psoriasis plaques.^23^

Like the IL-1 families, IL-36 requires N-terminal peptide cleavage to trigger pro-inflammatory activity through the action of proteases derived from neutrophil granules, cathepsin, elastase, and proteinase-3, increasing its biological activity by approximately 500 times.^18^, ^25^

The infiltrated neutrophils stain positive for cathepsin G, neutrophil elastase, and proteinase 3 in plaque psoriasis and pustular psoriasis lesions. In experimental studies, IL-36α was processed and activated by neutrophil elastase but not by cathepsin G, and its processing was prevented by serpin A1, a specific inhibitor of neutrophil elastase. IL-36γ was activated by cathepsin G, but not by elastase, and was inhibited by serpin A3, a specific inhibitor of cathepsin G. Cathepsin S derived from keratinocytes or fibroblasts is an enzyme that cleaves IL-36γ and transforms it into its active form, IL-36γ-Ser18. It is possible that IL-36γ-Ser18 induces hyperkeratosis and CXCL8 production and regulates the production of CXCL1, CXCL10, and CCL20 by keratinocytes (Fig. 2).^26^

Other enzymes participate in the endogenous processing of these cytokines, such as caspases (cysteine-aspartic proteases), proteolytic enzymes widely known for their role in controlling cell death and inflammation. Caspase 1 is essentially involved in gene expression and caspase 3 in IL-36γ release. Caspase-14 is expressed in the epidermis and plays a crucial role in the cornification and protection of the underlying layers of the skin.^23^, ^27^

in vitro stimulation of human keratinocytes with these cytokines increases gene expression of several chemokines to macrophages (CCL3, CCL4, CCL5, CCL2, CCL17, and CCL22), T lymphocytes (CCL20, CCL5, CCL2, CCL17, and CCL22), and neutrophils (CXCL8, CCL20, and CXCL1). In addition to keratinocytes, monocytes and myeloid dendritic cells (the latter ten times more than monocytes) express the IL-36 receptor.^28^ The presence of IL-36 members significantly increases the release of IL-1β and IL-6 by dendritic cells, human M2 macrophages, and Langerhans cells.^29^ The functional maturation of these cells is stimulated, and an autocrine system is maintained.^23^

Elevated levels of IL-36 receptor antagonists neutralize the pro-inflammatory reaction of IL-36 in plaque psoriasis and pustular psoriasis. The loss-of-function mutation of the IL-36RN gene leads to the release of IL-36 signaling, and these patients' keratinocytes produce higher levels of CXCL8 in response to IL-36.^23^

It has also been demonstrated that IL-36 is able to activate the vascular endothelium, leading to significant plasma extravasation, resulting in marked edema of the papillary dermis, extravasation of red blood cells, and other cells, such as eosinophils.^23^, ^24^

The expression of Th17/Th1-related cytokines, such as IL-17A, IL-22, IL-23p19, IFN-γ, and IL-18, is increased in plaque psoriasis when compared to GPP and normal skin. IL-17A induces IL-36 expression more intensely in psoriasis-derived human keratinocytes than in healthy keratinocytes.^23^

### Clinical manifestations

**Figure 3:**
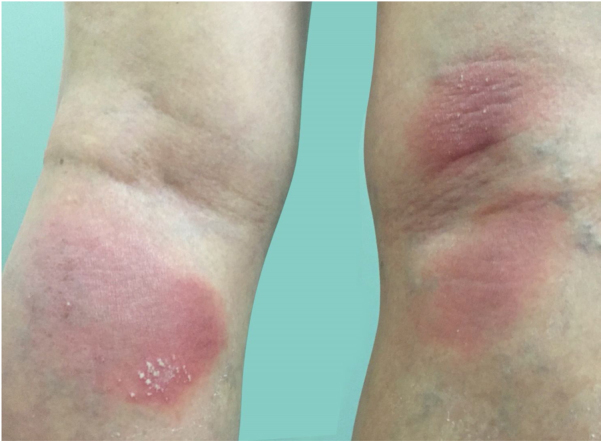
Early presentation of generalized pustular psoriasis.

**Figure 4:**
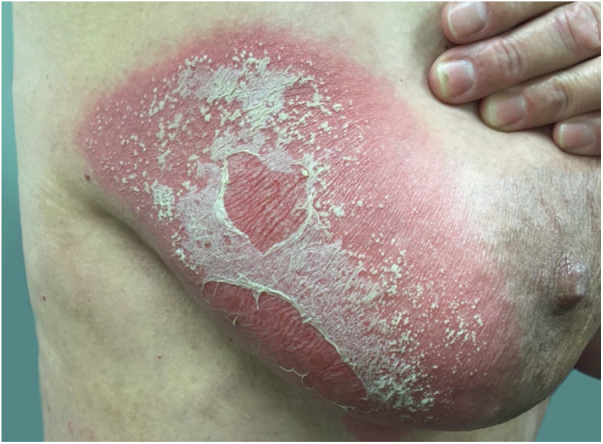
Lake of pus.

**Figure 5:**
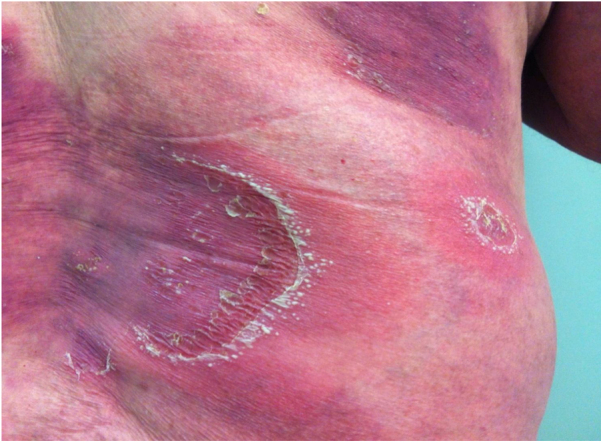
Eruptive presentation of generalized pustular psoriasis.

**Figure 6:**
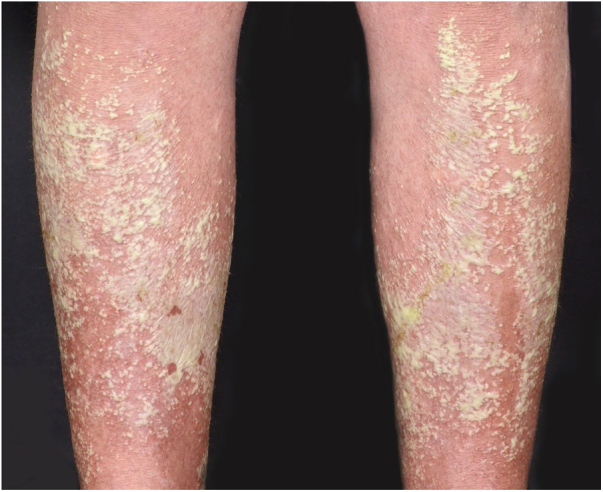
Generalized presentation affecting the lower limbs.

**Figure 7:**
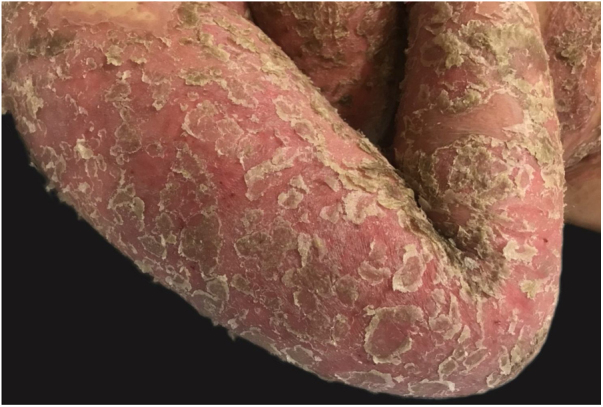
Severe condition affecting the upper limbs.

**Figure 8:**
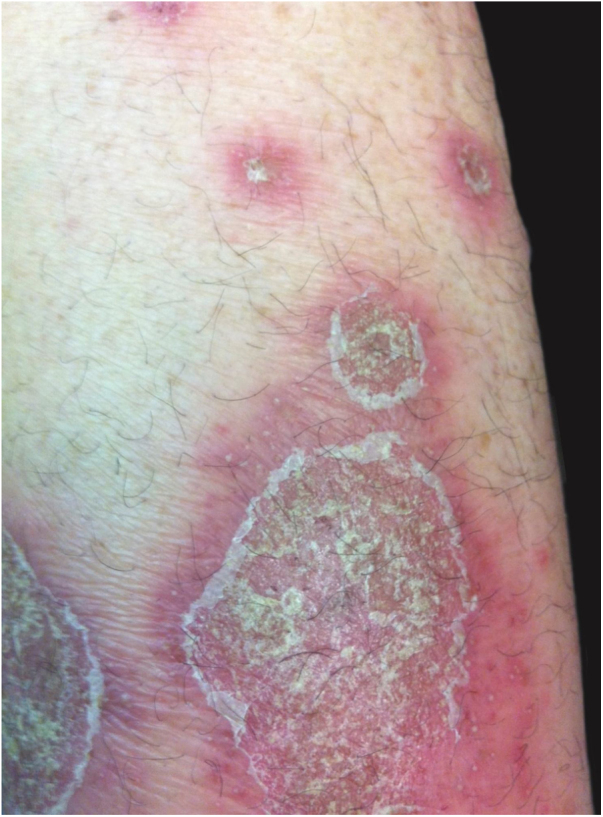
Pustular eruption over plaques of psoriasis vulgaris.

Symptoms onset tends to be abrupt and volatile, initially with the presence of erythema and edema of variable extent and degree, often affecting areas of large skinfols (Fig. 3). The skin is tender and painful. Within hours, tens to hundreds of sterile non-follicular pustules appear, which spread and may converge to form lakes of pus (Fig. 4). The eruption tends to be generalized, but there is a predilection for the trunk and proximal limbs (Figure 5, Figure 6, Figure 7), and the lesions may appear on apparently healthy skin or on previous plaque psoriasis lesions (Fig. 8). Pustular enanthema of the oral mucosa in periods of exacerbation and persistent geographic tongue may occur, and the lips may show desquamation and exulceration. Subungual pustules may be present.^30^

**Figure 9:**
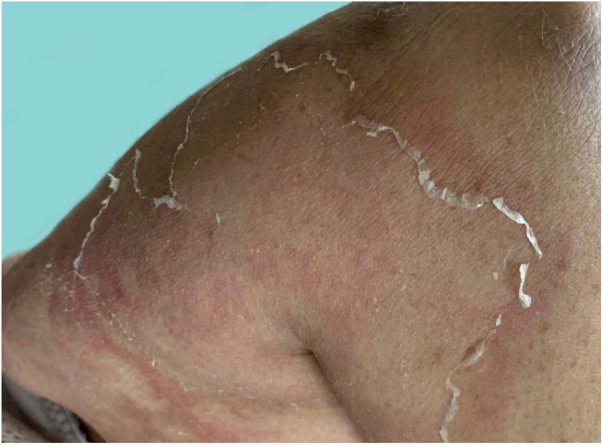
Residual lesions of pustular psoriasis.

As the condition progresses, the pustules desiccate, followed by scarlatiniform desquamation, leaving a smooth and shiny surface (Fig. 9). This usually persists for a few weeks, reverting to its previous condition or turning into erythrodermic psoriasis. Lesions tend to regress without sequelae. Rarely, hypertrophic scars or keloids may occur. Subsequent episodes of pustulation may occur, with time intervals ranging from weeks to years.^6^, ^30^

Systemic manifestations are frequent and often severe. The eruption may be accompanied by systemic symptoms such as fatigue, malaise, anorexia, nausea, tremors, and fever.^8^, ^31^ Overt ocular alterations, such as conjunctivitis, iritis, and uveitis may also be seen. Arthritis and osteomyelitis have also been reported.^30^

The condition can develop into potentially severe complications, such as bacterial superinfection, metabolic, hemodynamic, and thermoregulatory disorders, renal, hepatic, and cardiac failure, acute respiratory failure, neutrophilic cholangitis, pancreatitis, aseptic and hypovolemic shock, and death. These consequences are due to the breakdown of the skin barrier, vasodilation and hypoalbuminemia. Fever and poor general health status are usually associated. The occurrence of cholestasis secondary to neutrophilic cholangitis, as well as acute respiratory distress syndrome, if not diagnosed early enough, can also have a fatal outcome.^4^, ^32^

In the periods between GPP flare-ups, the patient tends to remain free from the systemic manifestations of the acute phase. These periods of respite can last weeks or years. Cases with associated plaque psoriasis tend to progress independently of the pustular crises.^30^, ^31^

In children with GPP, the course of the disease tends to be more benign, but severe and fatal outcome conditions are reported.^33^ Prompt and intensive management is mandatory, aiming to prevent severe complications such as bacterial superinfections, sepsis, metabolic, hemodynamic, and thermoregulatory disorders.^33^

Pustular psoriasis of pregnancy is a very specific manifestation of GPP, formerly called impetigo herpetiformis, and was first described by Hebra in 1872.^5^, ^34^ More frequent in the third trimester of pregnancy, it can also occur in early pregnancy, in the puerperium period, and even in the menstrual period. It usually resolves with the end of pregnancy but may recur in subsequent pregnancies with an earlier onset and a more severe clinical picture with each pregnancy. It is associated with an increased risk of fetal morbidity and mortality secondary to placental insufficiency.^5^, ^34^ Clinically, it consists of the eruption of small pustules over an erythematous base in a herpetiform arrangement and circinate disposition on the periphery of the plaques. In the center, the pustules gradually desiccate and crusts are formed. The eruption usually starts in the flexural areas of the skin, mainly on the inguinal region, with subsequent spreading.^5^, ^34^ Fever, diarrhea, and nausea may be present, as well as leukocytosis with neutrophilia, increased ESR, hypocalcemia, and hypoalbuminemia, which can lead to urinary and fecal incontinence, seizures, tetanism, and death.^35^ An early diagnosis and implementation of maternal and fetal vitality monitoring are essential. Although currently, the maternal prognosis is favorable, even in cases complicated by delirium, seizures, and tetanism secondary to hypocalcemia, the fetal prognosis is not as good. Fetal abnormalities, preterm birth, and neonatal death secondary to placental insufficiency can occur, even in well-controlled cases.^5^, ^34^

As well as in the case of GPP, the classification of pustular psoriasis of pregnancy is a matter of controversy. Although it is usually considered a variant of pustular psoriasis, some authors support its classification as a distinct entity related to pregnancy since most patients do not have a personal or family history of psoriasis, the disease resolves with the end of the gestational period and recurs in subsequent pregnancies.^5^, ^34^ On the other hand, reports of the development of GPP in women who had the specific form of the pregnancy-related disease years after remission favor the classification of the disease as a form of pustular psoriasis.^36^

### Diagnostic criteria

The 2017 European guidelines established the diagnostic criteria for GPP, including the presence of primary, sterile, non-follicular, and macroscopic pustules, affecting the non-acral skin; it may or may not occur in the presence of plaque psoriasis, being recurrent (more than one episode) or persistent (lasting more than three months).^37^

The 2018 Japanese guidelines established the following parameters for the diagnosis: a) systemic symptoms such as fever and fatigue; b) extensive or systemic flushing accompanied by multiple sterile pustules that can coalesce into lakes of pus; c) histopathology revealing subcorneal neutrophilic pustules, characterized as spongiform pustules of Kogoj; d) repeated recurrences of previous clinical and histopathological findings. The presence of four of the above criteria establishes the definitive diagnosis of GPP, and the presence of two or three criteria indicates diagnostic suspicion.^38^

### Triggering factors

Patients with plaque psoriasis may have pustular lesions over the plaques or far away, mainly due to aggravating factors such as infections, mainly of the upper respiratory tract, sunburns, local irritation by coal tar and anthralin, stress, pregnancy, or even an idiopathic trigger. The use of medications such as lithium, salicylate, tar, chloroquine, beta-blockers, the non-hormonal anti-inflammatory drug indomethacin, and, in particular, the use and discontinuation of systemic corticosteroids are classic aggravating factors of psoriasis.^39^

Hypocalcemia can be a cause or consequence of pustular psoriasis. Many studies suggest that vitamin D plays a role in epidermal cell differentiation and proliferation and cell adhesion requires cadherins, calcium-dependent molecules. There are cases of hyperparathyroidism and hypocalcemia with pustular psoriasis that reversed only with correction of the calcium disturbance.^32^, ^33^

Data from the European Registry of Severe Cutaneous Adverse Reactions (EuroSCAR) on the risk of severe cutaneous adverse drug reactions with 97 cases of generalized pustulosis and 1,009 controls showed that the drugs associated with GPP were pristinamycin, ampicillin or amoxicillin, quinolones, (hydroxy) chloroquine, sulfonamides, terbinafine, and diltiazem. The picture is difficult to differentiate from acute generalized exanthematous pustulosis (AGEP).^40^ There have been reports of cases triggered by terbinafine, in the form of *de novo* generalized pustulosis, classified as AGEP, the pustular transformation of plaque psoriasis, GPP, and palmoplantar pustulosis.^37^, ^41^

The use of interferon to treat a variety of conditions such as lymphomas and hepatitis has been associated with the onset and exacerbation of several forms of psoriasis, as well as topical imiquimod, which can increase interferon expression in treated skin and even at a distance.^42^

In a retrospective study of 102 patients with acute GPP, systemic glucocorticoids were considered the trigger in 44% of cases, and abrupt withdrawal of corticosteroids or use of depot corticoids as the only strategy for treating psoriasis or for treating another associated condition seems to be the most frequent situation.^43^

Choon et al. showed that acute infections are the triggering or exacerbating factor in 16% of patients, and 38.5% had antistreptolysin antibodies. Wang et al, in a study of 26 Chinese patients, showed that infections were responsible for triggering the disease in 73% of the cases.^43^

There are cases of GPP without previous manifestations of plaque psoriasis and in patients who have not been exposed to aggravating factors, and these cases should be the most probably related to the described genetic mutations.^9^ Phototherapy, ustekinumab, anti-TNFα, and even methotrexate, used to treat plaque psoriasis, can be aggravating and transforming factors for pustular psoriasis.^6^, ^44^

### Differential diagnosis

The clinical differential diagnosis is made with other pustular dermatoses, such as Sneddon Wilkinson subcorneal pustulosis, IgA pemphigus, amicrobial pustulosis of the folds, and especially AGEP. Differentiating between GPP and AGEP can be difficult, both clinically and histopathologically, especially when there is a history of drug exposure preceding the eruption. It is suggested that the absence of a personal or family history of psoriasis, rapid resolution with discontinuation of the suspected agents, as well as recurrence with re-exposure to the suspected drug associated with the presence of an eosinophilic infiltrate, significant edema of the superficial dermis, vasculitis, exocytosis of eosinophils, keratinocyte necrosis, and absence of psoriasiform alterations on histopathology favor the diagnosis of AGEP.^45^

On the other hand, a number of auto-inflammatory diseases can lead to sterile pustules. They originate from innate immunity alterations and from the excessive and uncontrolled activity of inflammasomes^46^ – protein complexes that control the production of pro-inflammatory cytokines. The normal function of inflammasomes allows the orderly production of IL-18 and, especially, of IL-1, necessary for the elimination of pathogens and cancer cells. The involvement of inflammasomes has been described in Sweet syndrome, vitiligo, hidradenitis suppurativa, atopic dermatitis, and psoriasis. A group of rare genetic syndromes associated with innate immune response activity with recurrent signs of systemic inflammation such as fever, which appear in childhood and may have cutaneous manifestations, are classified as monogenic autoinflammatory diseases. Examples are familial Mediterranean fever and Muckle-Wells syndrome. Auto-inflammatory diseases can have a variety of cutaneous manifestations, with widespread pustules, similar to pustular psoriasis, wheals, panniculitis, among others.^47^

A study on the several possible genetic mutations was carried out in nine children from six families who had neonatal sterile osteomyelitis, periostitis, and generalized pustulosis, and a genetic mutation was detected involving IL-1RN, the gene encoding the IL-1 receptor antagonist, which inhibits IL-1α and IL-1β, highly pro-inflammatory. This entity was called DIRA (Deficiency of the IL-1R Antagonist), described in newborn patients with a picture of sterile multifocal osteomyelitis, periostitis, discrete pustules to ichthyosiform pictures with oral ulcers, and ungual pitting. This aroused interest in studies of other dermatological conditions due to autoinflammation, such as SAPHO (synovitis, acne, pustulosis, hyperostosis, osteitis) syndrome and GPP.^17^

On the other hand, the mutation in the IL-36RN gene was originally reported in a study of nine families from Tunisia with GPP. DITRA (Deficiency of the IL-36R Antagonist) is the acronym for IL-36 receptor antagonist deficiency, found in familial or sporadic cases of GPP in Europe and Asia. It was initially described in cases of GPP, and later, less frequently, in PPPP, ACH, and AGEP, as well as in case reports and plaque psoriasis with pustular transformation. DITRA-associated conditions can occur from the newborn period to adulthood, and the clinical picture can be severe, with generalized pustulosis and systemic symptoms, progressing to complications of erythroderma, sepsis, and risk of death. The response to conventional treatments is insufficient, and patients fail to respond to the anti-TNF-α immunobiological in a short period of time. Reports demonstrate that anti-IL-17 and anti-IL-1 drugs seem to be more effective, and there are studies on anti-IL-36 for the treatment of these pustular forms (see below).^12^

## Histopathology

Similar to the findings of plaque psoriasis, in GPP there is hypergranulosis interspersed with parakeratosis (in correspondence with the suprapapillary epidermis), enlarged and regular rete pegs and suprapapillary thinning of the epidermis, increased vascularization of the dermal papilla, as well as a perivascular lymphoid infiltrate in the lower papillary dermis.^1^

**Figure 10:**
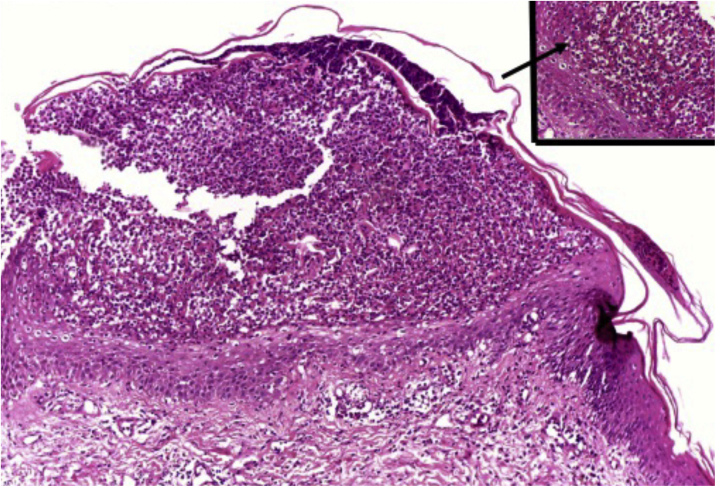
Pustular psoriasis: subcorneal pustule, of the spongiform type (inset, arrow). Hematoxylin & eosin, ×100 and ×400 (inset).

Clusters of neutrophils in the stratum corneum (Munro's microabscess), the presence of subcorneal pustules, and spongiform pustules of Kogoj are characteristic features (Fig. 10).^1^

Histopathologically, GPP must be differentiated from other dermatoses that may present with intraepidermal pustules, including Behcet's syndrome, bromoderma, iododerma, candidiasis, secondary syphilis, dermatophytosis, acropustulosis of infancy, drug reactions, insect bite reaction, impetigo, miliaria crystallina, pemphigus foliaceous, pyoderma gangrenosum, scabies, staphylococcal scalded skin syndrome, amicrobial pustulosis of the folds, and neonatal transient pustular melanosis.^30^

In cases of DITRA, the following peculiarities were detected: thin and compact stratum corneum, which is acidophilic with diffuse hypergranulosis between the pustules; spongiosis in the stratum spinosum; neutrophils migrating into the epidermis; basal keratinocytes with a regenerative aspect; marked edema of the papillary dermis with extravasation of red blood cells; eosinophils in the lymphohistiocytic inflammatory infiltrate in the lower papillary dermis.^22^

Histopathological differentiation from AGEP is difficult, and the presence of apoptotic keratinocytes and eosinophils favors the diagnosis of AGEP. The presence of tortuous vessels in the upper dermis seems to favor the diagnosis of GPP.^45^

It is important to remember that, when investigating pustular eruptions, direct immunofluorescence should be performed to rule out some types of pemphigus, such as those with intercellular IgA deposition or variants of pemphigus foliaceus and vulgaris with IgG anti-desmoglein 1 and 3.^48^

## Laboratory evaluation

Laboratory evaluation is necessary to assess the severity of the condition and diagnose possible complications. Laboratory alterations are related to the degree of inflammation and systemic involvement.

Hydroelectrolytic disturbances and hypovolemia, hypoalbuminemia, the elevation of liver enzymes and bilirubins, leukocytosis with neutrophilia and lymphopenia may occur. Elevation of inflammatory activity tests is frequently observed. Hypocalcemia can occur as a reflex of hypoalbuminemia, but it is usually asymptomatic and the ionic calcium level is normal.

It is recommended to perform a complete blood count, measurement of liver enzymes, urea, creatinine, C-reactive protein, erythrocyte sedimentation rate, calcium and albumin levels. Blood and urine cultures are required to rule out infections.^5^

The genetic tests discussed below are not routinely indicated, as their availabiliy is still limited and the cost is high.

## Genetic screening investigation

Three mutations account for less than 30% of GPP cases: IL-36RN, CARD14 and AP1S3. Although genetic testing for mutations is not routinely indicated due to difficult access and high cost, mutations in IL-36RN are increasingly used to aid in the diagnosis of GPP.

These mutations are associated with earlier age of onset in all pustular psoriasis subtypes, more severe progression to generalized inflammation, and different responses to treatment. Twelves et al. recommend that all patients with GPP onset before age 30 years should be screened for mutations in IL-36RN.^49^

## Treatment

In addition to being rare, GPP has a characteristic evolution in flare-ups with possible spontaneous remission, factors that make it difficult to carry out randomized clinical trials and make the development of standardized treatment regimens and treatment algorithms difficult.^9^

The extent and severity of GPP requires prompt intervention, hospitalization, and not infrequently, intensive care unit support.^9^

### Acitretin

Retinoids are the oldest systemic agents studied in the treatment of GPP. Etretinate was the first tested retinoid and its efficacy has been demonstrated in case series; however, it is not considered a therapeutic option, as it is no longer available.^9^

Acitretin had its efficacy demonstrated in retrospective studies^9^ conducted in Europe^4^ and Asia. Augey et al. evaluated 99 patients with GPP in 46 centers in France, in which acitretin was used in 89% of the patients as the first-line treatment, being considered by the authors as the most effective systemic treatment.^4^ In the second study, conducted by Choon et al., 102 patients with GPP were evaluated and 52 responded to systemic retinoids.

The recommended dose ranges from 0.75 to 1 mg/kg/day, and patients usually respond within 7 to 10 days.^50^ Due to its teratogenic potential, it should not be used by pregnant women or women of childbearing age.

### Methotrexate

The efficacy of methotrexate in GPP was demonstrated in two retrospective studies, the first with 24 patients and the second with 41 patients, being considered effective in 76.2% and 80% of the patients, respectively, but with different outcome analyses.^50^ The suggested doses are similar to those used in plaque psoriasis, ranging from 15 to 25 mg per week.

### Cyclosporine

Evidence supporting the use of cyclosporine comes largely from case reports.^50^ In a retrospective study, 66 patients were treated with cyclosporine in different centers in Japan, being considered effective in 71.2% of the patients.^50^

### Phototherapy

There is no evidence to recommend the use of phototherapy during the flare-up (acute phase) of GPP. There have been reports on the efficacy of PUVA phototherapy during the maintenance period, after the acute flare-up had been controlled.^9^

### Biologicals

#### TNF-alpha inhibitors

Since the approval of TNF-alpha inhibitors for plaque psoriasis, there have been several publications, mostly case reports and case series, reporting the efficacy of this therapeutic class in GPP.^50^

In a review published in 2018 by Boehner et al., 55 cases of GPP treated with anti-TNF alpha agents were identified in the literature, most of them (29 patients) with infliximab. Of the total number of treated patients, 58% had complete remission and 28% showed a partial response. It should be remembered that although anti-TNF alpha agents have been used in the treatment of GPP, they may be responsible for triggering flare-ups of acute GPP, which is considered a paradoxical reaction.^9^

#### Infliximab

The evidence to recommend infliximab use in the treatment of GPP comes from successful case reports and case series.^50^ A study carried out in Japanese patients with different types of psoriasis included seven patients with GPP, and response rates were around 70%.^50^ Its use in GPP was motivated by its fast action start. The National Psoriasis Foundation Pustular Psoriasis Treatment Guidelines (2012) considered infliximab the first-line treatment, thus placing etanercept and adalimumab as second-line treatments.^9^

#### Adalimumab

In an open-label, 52-week, multicenter study, Morita et al. evaluated the efficacy and safety of adalimumab in 10 Japanese patients with GPP, and the rate of complete remission was 50% in two weeks and 70% in 16 weeks. In the seven patients who had complete remission, the dose was gradually increased by 80 mg every two weeks. Although it is considered a second-line treatment in the National Psoriasis Foundation Pustular Psoriasis Treatment Guidelines (2012), it is the first anti-TNF alpha evaluated in a clinical trial that showed to be effective and safe in GPP management.^9^

#### Etanercept

It is the anti-TNF-alpha agent with the least reported evidence in the treatment of GPP, although case reports of therapeutic success have also been published.^50^ It appears as a second-line treatment in the National Psoriasis Foundation Pustular Psoriasis Treatment Guidelines (2012).^9^

#### Ustekinumab (Anti-IL-12 / IL-23)

Although cases of ustekinumab triggering GPP flare-ups have been reported,^9^ there are case reports and case series of therapeutic success, including its therapeutically successful use in the management of GPP triggered by TNF-alpha inhibitors.^50^ Arakawa et al. have published a case series of four cases of GPP refractory to several prior treatments (including anti-TNF alpha agents) that attained complete remission with the use of ustekinumab.^9^

#### Anti-IL-17

In recent years, open studies have been conducted evaluating the efficacy and safety of anti-IL-17 biologicals in the treatment of GPP. In an open-label study of 12 patients treated with secukinumab, 83% of the patients showed a good response.^9^ Brodalumab was evaluated in an open-label study that included 12 patients, with 83% improvement or remission seen within 12 weeks.^9^, ^50^ Similar results were also obtained with ixekizumab, in two studies that included patients with GPP.^9^ Results with Anti-IL-17 are promising, but studies with larger samples, comparison with a control group, and studies in non-Asian populations are still needed.^9^

#### Anti-IL-23

The initial promising results with anti-IL-17 have motivated the evaluation of anti-IL-23 biologicals, which also block the IL-23/Th17 axis, in the treatment of GPP. In an open-label study with guselkumab, ten patients with GPP were treated, with success rates of 50% in one week, and 100% in 52 weeks for the eight patients who completed the study.^9^

#### Targeted therapy against IL-1/IL-36 axis cytokines

The IL-1 family cytokines, particularly IL-36, play an important role in the immunopathogenesis of GPP,^9^ which has motivated researchers to evaluate therapies that target these cytokines.

Case reports have demonstrated the efficacy of anakinra (IL-1 receptor antagonist) and of canakinumab and gevokizumab (anti-IL-1 beta monoclonal antibodies).^9^, ^32^

New drugs that target the IL-36 receptor, anti-IL-36R, are under development (ANB019 and BI655130/spesolimab), and phase 1 results are promising. Phase 2 and 3 studies are already underway.^9^

### Treatment guidelines

No therapeutic agent for GPP has been approved in Europe and the USA to date. In Japan, the use of anti-IL-17 (secukinumab, ixekizumab, and brodalumab) and anti-IL-23 (guselkumab) immunobiological agents have been approved.^9^

The National Psoriasis Foundation (NPF) in 2012 published a GPP treatment guideline recommending acitretin, cyclosporine, methotrexate, and infliximab as first-line treatment options for GPP in adults.^9^

In cases of impetigo herpetiformis, the critical condition of the maternal-fetal binomial and the possibility of teratogenicity caused by drugs are challenges faced by dermatologists. Although drug interventions have been reported, the level of evidence is low and there are no controlled studies for the treatment of impetigo herpetiformis. To date, there are no specific treatment guidelines for impetigo herpetiformis.^34^

### Prognosis

GPP has a variable and unpredictable course and most patients have recurrent disease. The time interval between crises varies, and there may or may not be complete regression of the lesions between them. The severity of the crises can also vary in the same patient.

The emergence of pustules can occur without major signs of systemic inflammation or be associated with intense inflammatory conditions, with rapid progression and risk of death, requiring admission to an intensive care unit. Data on mortality rates are limited and between 3% and 7% of mortality in GPP cases have been reported.^33^

Identification of infection is often difficult due to the intense inflammatory process, making it a challenge to decide on the balance between the use of immunosuppressants and antibiotics. During crisis assessment, early identification of patients at higher risk and potential need of hospital admission should be sought. The careful monitoring of symptoms and clinical evolution is essential for early intervention, in an attempt to avoid severe complications.

As an incurable disease, the development of medication capable of preventing crises or even increasing the interval between them may improve the prognosis and quality of life of patients with GPP.^5^, ^49^

## Conclusion and future perspectives

GPP management remains a challenge in the clinical practice of dermatology, as it is a severe dermatosis for which there are few standardized treatment guidelines. The rarity of the disease and its characteristic evolution (flare-ups with periods of remission) limits the performance of better quality research protocols for therapy assessment.

Although scientific evidence is still scarce, the scenario seems to be changing due to advances in knowledge in the areas of genetics and immunology that have taken place in recent years and the advent of immunobiological therapy. The better genotypic and immunopathogenic characterization of the different pustular variants of psoriasis has allowed the development of new therapeutic targets.

## Financial support

None.

## Authors' contributions

Marcelo Arnone: Statistical analysis; approval of the final version of the manuscript; design and planning of the study; drafting and editing of the manuscript; collection, analysis and interpretation of data; effective participation in research orientation; intellectual participation in the propaedeutic and/or therapeutic conduct of the studied cases; critical review of the literature; critical review of the manuscript.

Renata Ferreira Magalhães: Statistical analysis; approval of the final version of the manuscript; design and planning of the study; drafting and editing of the manuscript; collection, analysis and interpretation of data; effective participation in research orientation; intellectual participation in the propaedeutic and/or therapeutic conduct of the studied cases; critical review of the literature; critical review of the manuscript.

Ricardo Romiti: Statistical analysis; approval of the final version of the manuscript; design and planning of the study; drafting and editing of the manuscript; collection, analysis, and interpretation of data; effective participation in research orientation; intellectual participation in the propaedeutic and/or therapeutic conduct of the studied cases; critical review of the literature; critical review of the manuscript.

André Luis da Silva Hirayama: Statistical analysis; approval of the final version of the manuscript; design and planning of the study; drafting and editing of the manuscript; collection, analysis, and interpretation of data; effective participation in research orientation; intellectual participation in the propaedeutic and/or therapeutic conduct of the studied cases; critical review of the literature; critical review of the manuscript.

## Conflicts of interest

Ricardo Romiti has conflicts of interest with the following laboratories, working as a consultant and/or speaker: Teva, Abbvie, Boeringer-Ingelheim, Janssen, Eli-Lilly, Leo Pharma, Novartis, PPfizer, UCB Biopharma. André L. S. Hirayama has conflicts of interest with the following laboratories, working as a speaker, researcher and in others as a consultant: Abbvie, Boeringer-Ingelheim, Novartis, Janssen, Eli-Lilly. Marcelo Arnone has conflicts of interest with the following laboratories, working as a speaker, researcher and in others as a consultant: Abbvie, Boeringer-Ingelheim,Novartis, Janssen, Eli-Lilly, Leo Pharma and UCB Biopharma. Renata F. Magalhães has conflicts of interest with the following institutions, working as a speaker and in others as a researcher: Abbvie, Novartis, Janssen-Cilag, Eli-Lilly and Unicamp.
